# The dispersal-related traits of an invasive plant *Galinsoga quadriradiata* correlate with elevation during range expansion into mountain ranges

**DOI:** 10.1093/aobpla/plab008

**Published:** 2021-06-16

**Authors:** Rui-Ling Liu, Ying-Bo Yang, Benjamin R Lee, Gang Liu, Wen-Gang Zhang, Xiao-Yan Chen, Xing-Jiang Song, Ju-Qing Kang, Zhi-Hong Zhu

**Affiliations:** 1 College of Life Sciences, Shaanxi Normal University, 710119 Xi’an, People’s Republic of China; 2 School for Environment and Sustainability, University of Michigan, Ann Arbor, MI 48109, USA; 3 National Engineering Laboratory for Resource Development of Endangered Crude Drugs in Northwest China, Shaanxi Normal University, 710119 Xi’an, People’s Republic of China

**Keywords:** Dispersal-related traits, elevation gradient, genetic variation, invasive plant, population differentiation

## Abstract

Detecting shifts in trait values among populations of an invasive plant is important for assessing invasion risks and predicting future spread. Although a growing number of studies suggest that the dispersal propensity of invasive plants increases during range expansion, there has been relatively little attention paid to dispersal patterns along elevational gradients. In this study, we tested the differentiation of dispersal-related traits in an invasive plant, *Galinsoga quadriradiata*, across populations at different elevations in the Qinling and Bashan Mountains in central China. Seed mass–area ratio (MAR), an important seed dispersal-related trait, of 45 populations from along an elevational gradient was measured, and genetic variation of 23 populations was quantified using inter-simple sequence repeat (ISSR) markers. Individuals from four populations were then planted in a greenhouse to compare their performance under shared conditions. Changing patterns of seed dispersal-related traits and populations genetic diversity along elevation were tested using linear regression. Mass–area ratio of *G. quadriradiata* increased, while genetic diversity decreased with elevation in the field survey. In the greenhouse, populations of *G. quadriradiata* sourced from different elevations showed a difference response of MAR. These results suggest that although rapid evolution may contribute to the range expansion of *G. quadriradiata* in mountain ranges, dispersal-related traits will also likely be affected by phenotypic plasticity. This challenges the common argument that dispersal ability of invasive plants increases along dispersal routes. Furthermore, our results suggest that high-altitude populations would be more effective at seed dispersal once they continue to expand their range downslope on the other side. Our experiment provides novel evidence that the spread of these high-altitude populations may be more likely than previously theorized and that they should thus be cautiously monitored.

## Teaser

In this study, we tested the differentiation of dispersal-related traits in an invasive plant *Galinsoga quadriradiata* across populations at different elevations in Qinling and Bashan Mountains, China. Our results suggest that the dispersal ability of *G. quadriradiata* decreases along elevational dispersal routes as a result of genetically based rapid evolution and phenotypic plasticity, challenging the common argument that dispersal ability of invasive plants increases along dispersal routes. Our findings provide new insight into how the dispersal ability of invasive plants may change along the elevational gradient. This is important for revealing the upward expansion dynamic of invasive plants in high mountains.

## Introduction

Invasive plants, which tend to spread uncontrollably and cause environmental or economic damage (Rejmánek *et al.* 2013), are increasingly colonizing into high-altitude areas of mountain ranges as a consequence of climate change (Alexander *et al.* 2016, 2018), land-use transformations and anthropogenic disturbances (Carboni *et al.* 2018). However, there has been relatively little attention paid to how mountains affect the upward expansion of invasive plants. It is often suggested that high mountain ranges are less likely to be invaded due to stressful abiotic environments (Körner 2007). Compared to lower altitude, plants are exposed to lower temperatures and shorter growing seasons in high-altitude area (Watermann *et al.* 2019), and soil nutrients may also be less abundant (Soethe *et al.* 2008; Drollinger *et al.* 2017). The importance of these factors is compounded by the fact that conditions can drastically change over a relatively small range of altitude (Becker *et al.* 2005). Invasive plants must therefore be able to acclimate to very different environments over small distances to move upslope.

Plants have been shown to do this via two main strategies: phenotypic plasticity and genetic adaptation (Liu *et al.* 2016a). Phenotypic plasticity refers to the ability of invasive plants to adjust their phenotype along environmental gradients and is important for supporting population persistence and continued spread across elevations (Alexander *et al.* 2016). Invasive plants usually demonstrate higher phenotypic plasticity compared to native species, which has been linked to their expansions in variable environmental conditions (Davidson *et al.* 2011; LaForgia *et al.* 2020). Phenotypic plasticity is typically important when environmental conditions are highly variable because it allows plants to acclimate within a relatively short timescale. Plant biomass allocation, height and number of flowers all demonstrate some degree of plasticity associated with elevation in mountain ranges (Frei *et al.* 2014; Ensslin and Fischer 2015). Other examples include leaf mass per area and leaf density, which usually decrease with elevation, while leaf thickness increases with elevation (Vitasse *et al.* 2014). It has thus been suggested that the invasion of some invasive plants benefits from phenotypic plasticity when expanding into higher elevations (Liao *et al.* 2016; Bustamante *et al.* 2018).

In contrast, genetic adaptation is more important over long timescales and occurs as a result of rapid evolution under novel natural selection pressure. Genetic differentiation among populations is an expected signal of adaptive response to changing environmental factors (Volis *et al.* 2015). Therefore, studies of population-level genetic differentiation of invasive species could offer insights into mechanisms of invasions (Ueno *et al.* 2015). For example, genetic differentiation may change during the expansion of invasive plants into high elevation (Bustamante *et al.* 2018), as has been observed for *Solidago canadensis*, for which genetic diversity decreased significantly with elevation (Alexander *et al.* 2009).

Although these mechanisms are likely to affect many traits related to plant fitness, range expansion is likely to be most affected by shifts in traits related to dispersal (Monty and Mahy 2010; Hargreaves *et al.* 2014; Huang *et al.* 2015; Tabassum and Leishman 2017). Spatial selection theory suggests that plants are sorted through space according to their dispersal ability or investment in dispersal (Thomson *et al.* 2018), and subsequent research has shown that individuals with greater dispersal ability accumulate at expansion range edges (Phillips *et al.* 2010; Shine *et al.* 2011; Andrade-Restrepo *et al.* 2019). This also implies that individuals with lower dispersal ability would form high-density populations in the center of the range. This is further supported by evidence showing that invasive plants usually experience decreased intraspecific competition and have high preadaptation ability at the invasion edge associated with low conspecific density (Alzate *et al.* 2020). Therefore, marginal populations with high dispersal ability will likely aggravate invasion (Phillips *et al.* 2010; Seale and Nakayama 2020). Moreover, high levels of dispersal can reduce the probability of inbreeding, thereby reducing the occurrence of deleterious genetic effects that might otherwise hinder invasion (Monty and Mahy 2010; Williams *et al.* 2019).

It is therefore expected that variation in traits related to dispersal will be found along the invasion routes of exotic plants (Rejmanek and Richardson 1996; Davidson *et al.* 2011; Kanapeckas *et al.* 2018), with traits associated with greater dispersal ability found at the invasive edge of the range. Detecting shifts in population-level traits of invasive plant species is thus an important tool for assessing invasion risks and predicting future spread (Estrada *et al.* 2016; Laeseke *et al.* 2020). However, few studies have tested whether the dispersal-related traits of invasive plants change along elevational gradients, and, if so, whether it is due to phenotypic plasticity or due to genetic adaptation (Monty and Mahy 2010; Steyn *et al.* 2017). Determining the source of the change in dispersal-related traits is important because evidence for phenotypic plasticity would indicate a greater risk of invasion associated with a higher ability to quickly acclimate to diverse environmental conditions, whereas evidence for genetic variation would instead suggest that a greater risk of invasion associated with a higher genetic diversity.

In this study, we investigated how dispersal-related traits of invasive *Galinsoga quadriradiata* vary along an elevational gradient in a high mountain range in central China, and whether such changes are attributable to genetic differentiation or phenotypic plasticity. Specifically, we addressed the following questions: (i) How do population-level traits of *G. quadriradiata* related to dispersal vary across an elevational gradient? (ii) Are these changes attributable to phenotypic plasticity, genetic adaptation or a combination of both? (iii) What do these changes suggest about the future expansion of *G. quadriradiata* in the mountains of central China?

## Materials and Methods

### Study organism

*Galinsoga quadriradiata* (Asterales: Asteraceae) is an annual herbaceous plant originating in Central and South America. It is a harmful agricultural invasive weed, mainly established in moist, warm temperate and subtropical zones around the world on abandoned land or farmland (Liu *et al.* 2016b). It can reduce agricultural production by approximately 50 % (Kabuce and Priede 2010). Since its first report on Lushan Mountain in Jiangxi Province in 1979, it has been found across all suitable climatic areas in China (Yang *et al.* 2018). Due to a lack of competitive and colonization ability in natural communities, it is usually considered a relatively weak invader (Liu *et al.* 2018). However, its influences on natural and agricultural ecosystems are significant due to its considerably large seed yield (approximately 46 000 fertile seeds per square meter) and high dispersal potential attributed to light seed mass which covered with hairs (Liu *et al.* 2016b). *Galinsoga quadriradiata* has recently started to expand towards the northern and western regions of China, which are at relatively high altitudes (Yang *et al.* 2018). This includes the Qinling and Bashan Mountains, high peaks that act as major barriers preventing the further dispersal of exotic species from the coastal areas of eastern and southern China. Large populations are already widely established on the southern slopes, threatening the conservation of biodiversity as well as agricultural production in this region (Liu *et al.* 2016b).

### Study location

The Qinling and Bashan mountain ranges in central and south-western China (30˚5'–34˚59'N, 102˚54'–112˚4'E, about 222 300 km^2^) are characterized by complex topography and distinct environmental conditions between the northern and southern regions (Wang *et al.* 2017). The highest peaks in the Qinling and Bashan Mountains are at 3767 and 3105 meters above sea level (m asl), respectively. The mountains serve as natural barriers between the southern subtropical and the northern warm temperate regions of central China, and also as a boundary between the Palearctic and Oriental Regions in eastern Asia. In the Qinling Mountains, areas lower than 1000 m asl on south-facing slopes are dominated by a subtropical climate while the areas above 1000 m asl on south-facing slopes and all elevations on north-facing slopes are more temperate. The Bashan Mountains are approximately 50 km south of the Qinling Mountains, extend from east to west in parallel with them (Zhan *et al.* 2009), and are dominated by a subtropical climate. In the Qinling Mountains, the average annual temperature is 11–13 °C and the average annual precipitation is between 590 and 764 mm (Zhao *et al.* 2014). The average annual temperature in the Bashan Mountains ranges from 14.5 to 16.5 °C, and average annual precipitation is between 800 and 1400 mm (Li *et al.* 1990).

### Field survey

**Figure 1.**
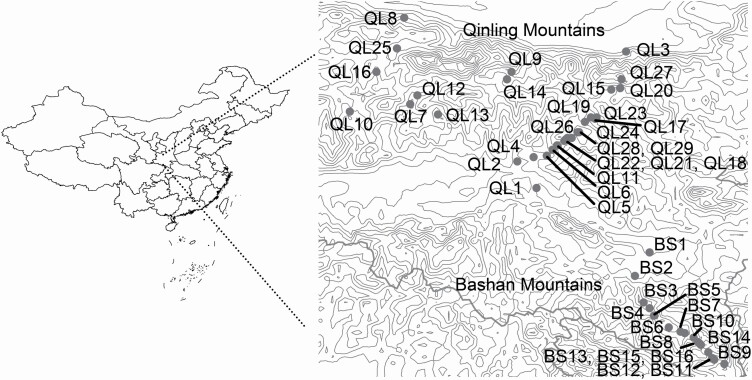
Map of the elevational populations of *G. quadriradiata* investigated in the Qinling and Bashan Mountains.

In July 2015, we conducted a field survey across both mountain ranges. In total, 45 populations of *G. quadriradiata* were surveyed along an elevational gradient ranging from 220 to 2128 m asl (Fig. 1; Table 1). Each population was separated by at least 150 m in altitude. In each population, we randomly chose 4–20 mature individuals to collect seed and leaf samples from. The number of mature seeds per capitulum (hereafter NSC) was counted after randomly choosing at least 5 capitula per individual. All seeds were air-dried and loosely stored in envelopes at 4 °C until measurement. We also collected approximately 8–16 young leaves per plant. The collected leaves were quickly dried in a plastic bag with silica gel and then stored in a −80 °C freezer until use.

**Table 1.** The locations of the elevational populations of *Galinsoga quadriradiata*. The last column showed the populations used for genetic diversity analysis.

Population	Longitude (°)	Latitude (°)	Elevation (m)	Mountains	Genetic
BS1	108.98	32.65	223	Bashan	
BS2	108.88	32.49	370	Bashan	Y
BS3	108.94	32.31	436	Bashan	Y
BS4	108.98	32.27	504	Bashan	Y
BS5	109.01	32.22	646	Bashan	Y
BS6	109.11	32.14	691	Bashan	
BS7	109.19	32.11	859	Bashan	Y
BS8	109.22	32.1	890	Bashan	Y
BS9	109.49	31.89	1005	Bashan	Y
BS10	109.28	32.07	1185	Bashan	Y
BS11	109.42	31.92	1248	Bashan	Y
BS12	109.41	31.93	1321	Bashan	Y
BS13	109.31	32.04	1513	Bashan	Y
BS14	109.38	31.97	1594	Bashan	Y
BS15	109.32	32.03	1756	Bashan	Y
BS16	109.33	32.02	1947	Bashan	Y
QL1	108.21	33.09	414	Qinling	Y
QL2	108.08	33.27	523	Qinling	
QL3	108.82	34.02	565	Qinling	
QL4	108.19	33.3	581	Qinling	Y
QL5	108.28	33.31	680	Qinling	
QL6	108.32	33.35	829	Qinling	Y
QL7	107.35	33.66	839	Qinling	
QL8	107.31	34.25	855	Qinling	
QL9	108.04	33.88	860	Qinling	
QL10	106.94	33.61	896	Qinling	
QL11	108.35	33.38	916	Qinling	
QL12	107.4	33.72	985	Qinling	
QL13	107.54	33.59	1041	Qinling	
QL14	108.01	33.83	1048	Qinling	Y
QL15	108.72	33.76	1060	Qinling	Y
QL16	107.12	33.88	1097	Qinling	
QL17	108.61	33.56	1163	Qinling	
QL18	108.4	33.41	1203	Qinling	Y
QL19	108.58	33.57	1229	Qinling	
QL20	108.78	33.77	1236	Qinling	Y
QL21	108.41	33.42	1262	Qinling	
QL22	108.42	33.43	1307	Qinling	
QL23	108.62	33.57	1343	Qinling	
QL24	108.54	33.54	1349	Qinling	Y
QL25	107.26	34.04	1454	Qinling	
QL26	108.45	33.43	1563	Qinling	Y
QL27	108.79	33.83	1725	Qinling	
QL28	108.5	33.47	2080	Qinling	
QL29	108.49	33.47	2128	Qinling	

### Measurement of dispersal-related traits

For each plant from the field survey, we calculated NSC and measured the combined weight of one hundred seeds (HSW). To calculate HSW, we randomly selected 100 ripe seeds from the collected seeds of each population and weighted them. At least five replicates were made for each population. We used WinSEEDLE Pro (WinSEEDLE™, Régent Instruments Inc., Québec, QC, Canada) to measure seed length, pappus length and pappus width. Each time, we randomly selected 30 ripe seeds from an elevational population and scanned and analysed them. We repeated this 10 times for each population.

Dispersal ability of wind-dispersed Asteraceae diaspores, such as *G. quadriradiata*, is typically approximated by morphological characteristics (Matlack 1987; Monty and Mahy 2010; Seale and Nakayama 2020). Here, we used plume loading (mass–area ratio, MAR), a morphological characteristic commonly associated with dispersal ability (Huang *et al.* 2015). Mass–area ratio (MAR) can be calculated as


$$\begin{array}{l} MAR=\frac{m}{\pi R^{2}}, \end{array}$$

where *m* is the seed mass and *R* is the pappus radius which is calculated as half of the pappus width. Mass–area ratio has been shown to be a reliable indicator of the dispersal ability of other wind-dispersed Asteraceae (Matlack 1987; Meyer and Carlson 2001), so it should also be representative of dispersal ability of our study species. Low MAR values (i.e. lighter seed with larger pappus) therefore indicate greater dispersal potential (Augspurger and Franson 1987).

### Genetic differentiation

We used the inter-simple sequence repeat (ISSR) markers to analyse the genetic diversity of *G. quadriradiata* populations. Total DNA was isolated using the CTAB method (Gawel and Jarret 1991), and diluted in sterilized Millipore water. Inter-simple sequence repeat primers were synthesized (by Shanghai Sangon Biological Engineering Technology & Service Co., Ltd) according to the primer set published by the University of British Columbia (UBC) (http://www.michaelsmith.ubc.ca/services/NAPS/Primer_Sets/Primers_Oct2006.pdf). Seven primers (UBC 816, UBC 817, UBC 818, UBC 826, UBC 847, UBC 855 and UBC 857) were used to amplify the 233 samples from 23 of the 45 populations. The polymerase chain reaction (PCR) system for ISSR analysis was as follows: total volume 25 µL; 12.5 μL 2 × Es *Taq* MasterMix (Dye), 1 μL 10 μmol L^−1^ primer, 1 μL 50 ng μL^−1^ DNA, 10.5 μL ddH_2_O. All reactions were performed on a gradient PCR instrument (Agilent SureCycler 8800). The PCR procedure was as follows: pre-degeneration at 94 °C for 2 min, degeneration at 94 °C for 30 s, annealing at 50.5–55 °C for 30 s (annealing temperature depends on different primer), extension at 72 °C for 1 min, 6 cycles; degeneration at 94 °C for 30 s, annealing at 50.5–55 °C for 30 s (annealing temperature depends on different primer), extension at 72 °C for 1 min, 32 cycles; final extension at 72 °C for 3 min and preservation at 4 °C. The amplification products were separated in 2 % agarose gel (containing 0.5 mg mL^−1^ ethidium bromide) in 1 TBE (Tris-Borate-EDTA), and the separated bands were visualized under UV light by using an Electrophoresis Documentation and Analysis System 120 (Eastman Kodak Company). DL2000 ladder (Dongsheng Biotech Ltd) was used as DNA molecular weight markers.

### Greenhouse experiment

To explore the influence of genetic differentiation on dispersal ability of different populations in a common environment on the basis of field survey, we randomly selected populations from four elevations. In late April 2016, seeds from the four populations (BS1 (223 m asl), QL5 (680 m asl), QL22 (1307 m asl) and BS15 (1756 m asl); Table 1) were sown in nursery pots in a greenhouse (at 430 m asl) located on a plain on the northern side of the Qinling Mountains. The environmental condition of the QL5 population was most similar to that of the greenhouse. Three weeks later, when the seedlings were approximately 5 cm in height, they were carefully transplanted into plastic pots (diameter 12 cm, height 10 cm). The pots were filled with a 1:1 mixture of soil and sand using soil collected from a forest plantation next to the greenhouse. The total nitrogen and total phosphorus concentrations of the mixed soil were 3.02 and 0.65 mg g^−1^, respectively. Eight replicate plants from each population were grown, yielding a total of 32 pots. Pots were randomly arranged in an 80-m^2^ greenhouse, and the positions of the pots were changed at random every 2 weeks. Seedlings were watered daily and were replaced if mortality occurred within 1 week of the start of the experiment. The plants began to bloom in early June and the seeds began to disperse in late June. We collected mature seeds every 2 days until all plants no longer yielded seeds. All seeds were stored in envelopes and air-dried under laboratory conditions until measurement. We then analysed seed dispersal-related traits using the same method as described above for the field survey.

### Data analyses

We constructed linear mixed-effects models to evaluate the effects of elevation on diaspore-related traits (HSW, NSC, seed length, pappus length, pappus width and MAR), using the packages ‘lme4’ and ‘lmerTest’ in R-3.5.3 (R Development Core Team 2019). Elevation and mountain (Qinling and Bashan) were used as the fixed and random factors, respectively, in the model.

Only distinct, reproducible and well-resolved PCR fragments were included in the statistical analysis for genetic differentiation. Inter-simple sequence repeat bands were scored as presence (1) or absence (0) characters, to construct the binary matrix. To compare the amount of total genetic variation partitioned within and among populations, three methods were employed, namely the hierarchical analysis of molecular variances (AMOVA), the analysis of Shannon’s diversity (Qian and Ge 2001) and Nei’s analysis of genetic diversity (Nei 1987). Genetic data were analysed in GenAlEx 6.502 (Peakall and Smouse 2012). Genetic diversity (i.e. the percentage of polymorphic loci, the Shannon’s information index and the expected heterozygosity) was calculated. The percentage of polymorphic loci (PPL) is an indicator of genetic polymorphism, with higher values indicating a higher proportion of polymorphic loci, and thus greater genetic variability. The Shannon’s information index (*I*) is usually used as an evaluation index, with higher values reflecting higher genetic diversity. Expected heterozygosity (*H*_e_) is often used to measure the genetic diversity of a population, with higher values indicating richer the genetic diversity.

Linear mixed-effect models were then constructed to test for associations between genetic diversity and elevation (fixed factor) for the field seedlings, using mountain (Qinling and Bashan) as a random factor. We then constructed the phylogenetic tree of UPGMA based on Nei’s genetic distance using MEGA-X (Kumar *et al.* 2018). For the greenhouse experiment, we performed a linear regression analysis to evaluate the effects of elevation on the four dispersal-related traits (seed length, pappus length, pappus width and MAR) using the package ‘multcomp’ in R-3.5.3 (R Development Core Team 2019). We also performed a one-way ANOVA analyses and Tukey’s multiple comparison tests to compare the MAR of the four populations in the greenhouse experiment and the field survey.

## Results

### Phenotypic trait variation in the field survey

**Figure 2.**
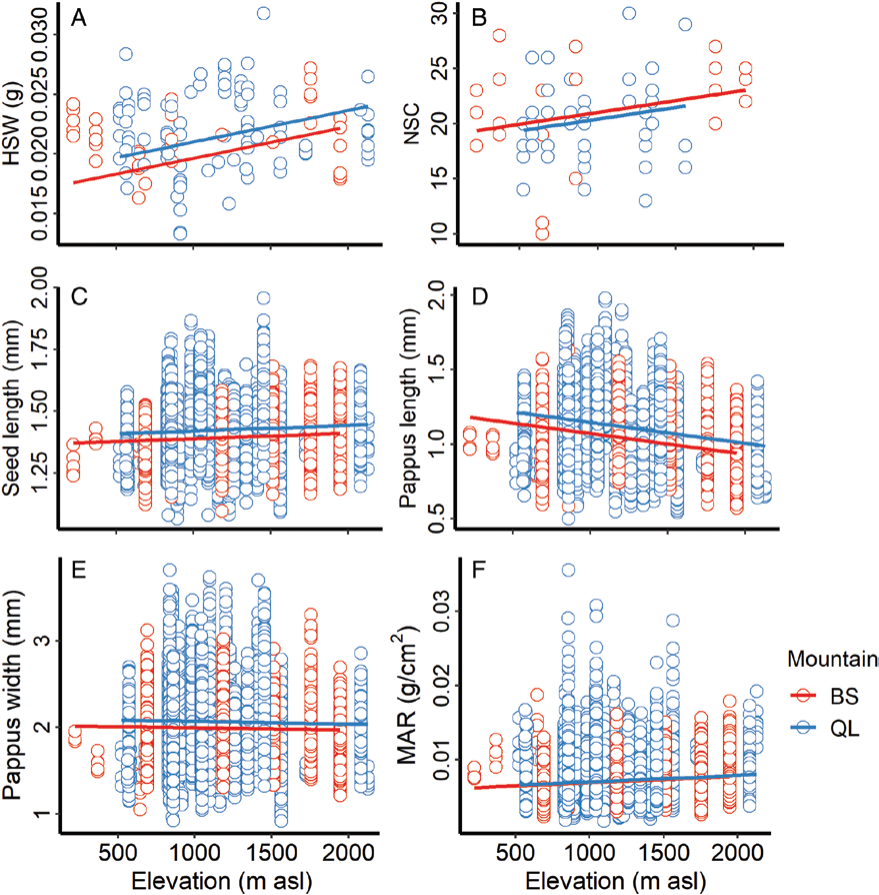
The linear mixed-effect model of seed dispersal-related traits (HSW, NSC, seed length, pappus length, pappus width and MAR) and elevation in the field survey. Fixed factor, Elevation; random factor, Mountain. HSW, the weight of one hundred seeds; NSC, the number of mature seed per capitulum; MAR, mass–area ratio.

The results of the linear mixed-effect models revealed that elevation has significant effects on HSW, NSC, seed length, pappus length, pappus width and MAR (Table 2; **see****Supporting Information—Table S1**). HSW (Fig. 2A), NSC (Fig. 2B), seed length (Fig. 2C) and MAR (Fig. 2F) showed significant positive correlations with elevation while pappus length (Fig. 2D) and pappus width (Fig. 2E) were significantly negatively correlated with elevation.

**Table 2.** The results of linear mixed-effect models on MAR, HSW (weight of one hundred seeds), NSC (number of mature seed per capitulum), seed length, pappus length and pappus width using elevation and mountain as fixed and random factors, respectively. *P < 0.05; **P < 0.01; ***P < 0.001; ^ns^*P* > 0.05.

Dependent variable	Fixed effect	Estimate	SE	*t*	*P*	Signif. Codes
MAR	Intercept	0.006	1.35 × 10^–4^	44.753	<0.001	***
	Elevation	8.83 × 10^–7^	9.27 × 10^–8^	9.526	<0.001	***
HSW	Intercept	0.018	6.94 × 10^–4^	25.46	0.02	*
	Elevation	2.65 × 10^–6^	1.05 × 10^–7^	25.15	<0.001	***
NSC	Intercept	18.59	1.096	16.961	<0.001	***
	Elevation	0.002	9.18 × 10^–4^	2.318	0.0231	*
Seed length	Intercept	1.382	0.016	84.45	0.004	**
	Elevation	2.27 × 10^–5^	3.50 × 10^–6^	6.48	<0.001	***
Pappus length	Intercept	1.249	0.039	32.3	0.0143	*
	Elevation	–1.4 × 10^–4^	6.79 × 10^–6^	–20.69	<0.001	***
Pappus width	Intercept	2.061	0.04	50.969	0.003	**
	Elevation	–2.75 × 10^–5^	1.25 × 10^–5^	–2.193	0.028	*

### Genetic diversity

**Figure 3.**
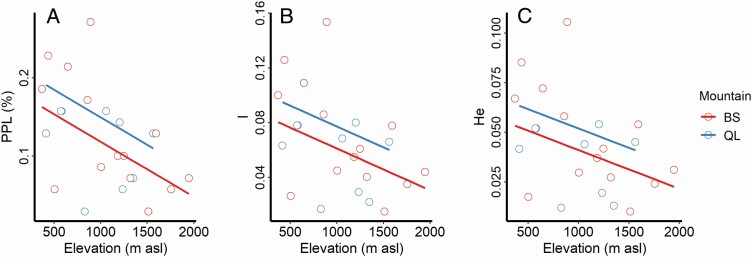
The linear mixed-effect model of genetic diversity (PPL, *I* and *H*_e_) and the elevation of the field population. Fixed factor, Elevation; random factor, Mountain. PPL, percentage of polymorphic loci (%); *I*, Shannon’s information index; *H*_e_, expected heterozygosity.

A total of 70 polymorphic loci were obtained from the ISSR analysis. Genetic variation between populations accounted for 81 % of total genetic variation while intrapopulation variation accounted for the remaining 19 % (Table 3). PPL, *I* and *H*_e_ were significantly negatively correlated with population elevation (Fig. 3; Table 4; **see****Supporting Information—Table S2**), indicating that genetic diversity decreases at higher altitudes.

**Table 3.** The result of AMOVA analysis for evaluating genetic variation from among and within populations. The AMOVA analysis was significant after 999 permutation tests (PhiPT = 0.809, *P* = 0.001).

Source	df	SS	MS	Est. Var.	%
Among pops	22	1449.225	65.874	6.368	81 %
Within pops	210	316.431	1.507	1.507	19 %
Total	232	1765.657		7.875	100 %

SS, sum of squared deviation; MS, mean square deviation.

**Table 4.** The results of linear mixed-effect models on PPL, *I* and *H*_e_ using elevation and mountain as fixed and random factors, respectively. *P < 0.05; **P < 0.01; ***P < 0.001; ^ns^*P* > 0.05.

Dependent variable	Fixed effect	Estimate	SE	*t*	*P*	Signif. codes
PPL	Intercept	0.204	0.016	12.93	0.046	*
	Elevation	−7.05 × 10^–5^	1.46 × 10^–6^	–48.47	<0.001	***
*I*	Intercept	0.1	0.008	12.28	0.048	*
	Elevation	−3.05 × 10^–5^	7.88 × 10^–7^	–38.71	<0.001	***
*H*_e_	Intercept	0.066	0.005	12.05	0.049	*
	Elevation	−1.95 × 10^–5^	5.44 × 10^–7^	–35.91	<0.001	***

**Figure 4.**
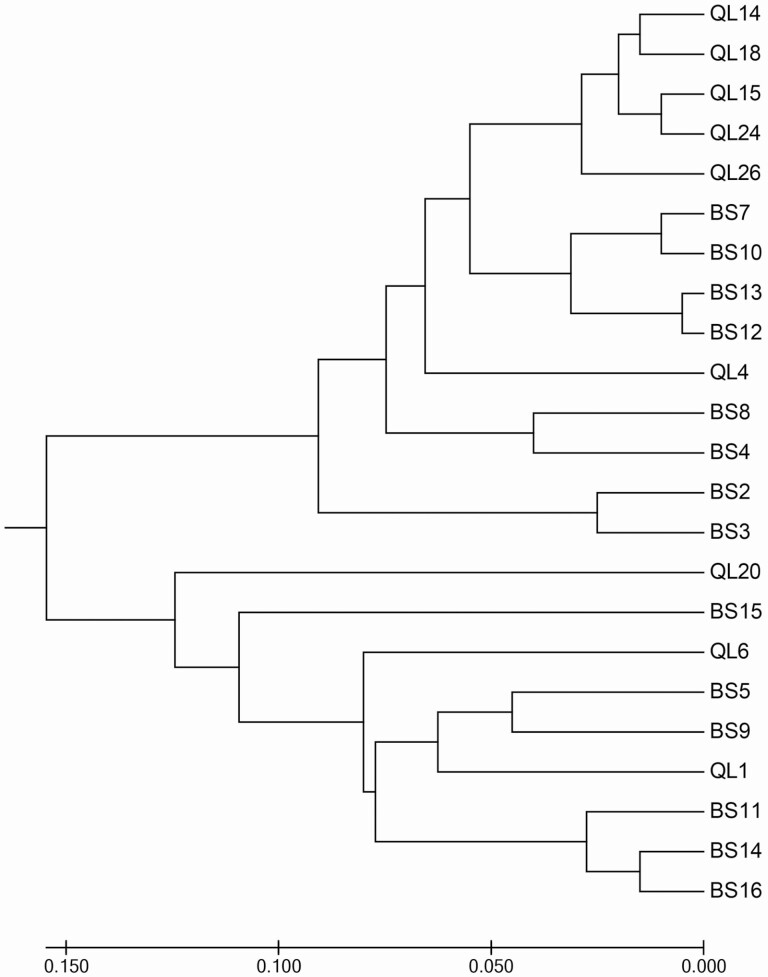
UPGMA cluster analysis of 23 populations of *G. quadriradiata* based on Nei’s genetic distances.

The sum of the branch length of the optimal phylogenetic tree was 1.32 (Fig. 4). Populations were genetically clustered into two groups, each of which not only contained sites from both mountains, but also contained sites spanning multiple elevations. Smaller population clusters were typically composed of populations that had similar elevations or that were geographically closed to each other.

### Greenhouse experiment

**Figure 5.**
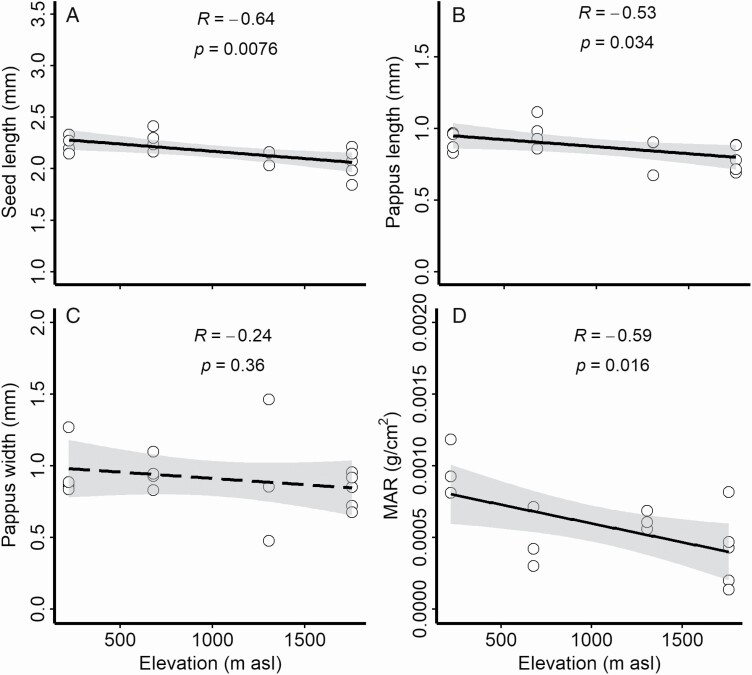
The linear relationship between seed dispersal-related traits and the elevation of the source population in the greenhouse experiment. MAR, seed mass–area ratio.

The interpopulation trends of dispersal-related traits described above for the field survey were differed substantially from the results from the greenhouse study. Seed length (Fig. 5A), pappus length (Fig. 5B) and MAR (Fig. 5D) were significantly negatively correlated with the elevation of the seed source population. Pappus width was not significantly correlated with the elevation of the seed source population (Fig. 5C).

## Discussion

Elevational variation associated with seed dispersal-related traits of invasive species has not yet been fully explored, creating a knowledge gap of how phenotypic plasticity and genetic diversity affect the invasive success of plants at high altitudes. In this study, we examined the phenotypic plasticity and genetic diversity of dispersal-related traits in invasive *G. quadriradiata* in the mountains of central China, and investigated whether dispersal-related traits variation in this species is attributable to genetic differentiation, plasticity or some combination thereof. We found that many trait values were significantly associated with elevation in our field survey, with plants from higher-elevation populations tending to have larger seeds with smaller pappi (feathery protusions which aid in dispersal). Furthermore, these trends were associated with reductions in genetic diversity at high elevations, suggesting that adaptation is largely responsible for the observed patterns. However, some of contradicted results from our greenhouse study showed greater dispersal-related traits of higher-elevation population in unstressed environment, suggesting that trait plasticity could still play an important role in the range expansion of invasive species in mountain ranges.

### The clinal trend of dispersal-related traits along the elevational route

Spatial selection theory hypothesizes that dispersal phenotypes are spatially separated and that only the best dispersers will accumulate towards the range front (Shine *et al.* 2011). Evidence to support this theory has been demonstrated in many invasive plants (Monty and Mahy 2010; Huang *et al.* 2015) as well as in evolutionary simulation models (Dytham 2009). This theory is also consistent with genetic differentiation theory (Travis and Dytham 2002; Alford *et al.* 2009; Andrade-Restrepo *et al.* 2019), which predicts high dispersal ability in individuals near the forward edge of the expanding range. In mountain ranges, where the range edge is at high elevation, we would thus expect to find correlations between dispersal ability and elevation.

For wind-dispersed Asteraceae seeds, dispersal ability is often approximated by measuring seed MAR, which is correlated with terminal seed velocity (Monty *et al.* 2008; Monty and Mahy 2010; Seale and Nakayama 2020). Higher MAR values have typically been found to indicate lower dispersal ability (Huang *et al.* 2015), so, following spatial selection theory, one would expect that invasive plants at high elevation should have lower MAR than those closer to the center of their range.

We found the opposite trend in our field survey, with *G. quadriradiata* seeds exhibiting positive correlations between MAR and elevation (Fig. 2F) and negative correlations between elevation and traits related to pappus size (Fig. 2D and E). In contrast to spatial selection theory, this suggests that this species has a diminished dispersal ability at high elevations. If this is true, it would mean that mountain ranges slow the invasion of *G. quadriradiata* by negatively affecting dispersal, a novel finding for high-elevation invasion dynamics.

Still, it is important to note these trends could instead indicate that low MAR and large pappi are not as advantageous in high-elevation environments as they are in other systems. For example, increased wind strength and occurrence of updrafts associated with mountain ranges might allow plants to achieve the same dispersal distance with relatively smaller investments in dispersal-related traits (Matlack 1992). To this point, seed mass has been found to increase with elevation for plants in the genera Solidago (Hirano *et al.* 2017), and more broadly in plants that span broad elevational gradients in Australia (Satyanti *et al.* 2018). In addition, invasive plants richness usually increases with the increased disturbance intensity of anthropogenic disturbances in different kinds of habitats (Alston and Richardson 2006; Pinke *et al.* 2011). It is probably because the seed dispersal and seedling recruitment of invasive plants usually can be promoted by heavy traffic and soil disturbance (Kröel-Dulay *et al.* 2018; Lemke *et al.* 2019). Therefore, the trends we found in our field survey could instead be indicative of successful adaptation to montane environments and different kind of disturbance, which may imply greater invasion success.

We speculate that our results are more supportive of the first hypothesis, particularly because a trade-off between dispersal ability and seed mass has broad support in the literature (Cappuccino *et al.* 2002; Huang *et al.* 2015; Tabassum and Leishman 2017; DiTommaso *et al.* 2018). Although the NSC increased with altitude (Fig. 2B), the number of capitulum and total seed production per plant decreased (Liu *et al.* 2016b) suggesting that overall fecundity is reduced at high elevation. However, a more detailed study on the relationship between the traits we studied and dispersal success at our study site is required before making further conclusions.

### Population genetic differentiation

We found strong support that the observed interpopulation trait variation across elevation was driven by genetic variation rather than by phenotypic plasticity. We also found that the genetic diversity of populations decreased with increasing elevation (Fig. 3). Together, our results suggest that genetic differentiation occurs during the expansion of *G. quadriradiata* along elevation and that the resulting genetic variation results in more homogeneous communities. These results agree with previous studies of population-level genetic characteristics, which have demonstrated that marginal populations exhibit lower genetic diversity and higher genetic differentiation than central populations (Lammi *et al.* 1999; Eckert *et al.* 2008; Excoffier and Ray 2008). Theoretically, this is expected to occur when two key genetic parameters, effective population size (*N*_e_) and the rate of gene flow (*m*), are strongly influenced by the demography and spatial distribution of populations, with optimal parameter values in central populations and less optimal values in marginal populations (Excoffier and Ray 2008). Our results agree with the theoretical predictions of genetic differentiation and intrapopulation diversity, so we conclude that similar dynamics are likely to have occurred for our study species.

The phylogenetic tree showed that the smallest group is made up of different populations from the same mountain (Fig. 4). However, as the number of branches decreased, the result of clustering became chaotic. Populations from the Qinling Mountains were clustered with others from the Bashan Mountains, suggesting that there are high rates of gene flow among the populations of the two large mountains. It seems unlikely that gene flow is occurring naturally between the two mountain ranges given the distances involved, so we instead speculate that these similarities could be the result of human activities. This region of China has recently experienced increased development with a greater number of roads and increasing traffic (Liu *et al.* 2016b; Smith *et al.* 2020). The additional disturbance caused by the increased construction and number of visitors (Alston and Richardson 2006; Pauchard *et al.* 2009), as well as the additional dispersal route created by people who may incidentally disperse seeds between the two mountains (Liu *et al.* 2016b), could both lead to the phylogenetic results that we observed in this study.

### Mechanisms for changing dispersal traits

Abiotic filtering can inhibit the invasion process of invasive species in mountain ranges because of the time that is typically necessary for genetic adaptation (Marini *et al.* 2012). Plasticity acts at the level of the individual and thus can enable organisms to adapt and survive in rapidly changing environments (Fox *et al.* 2019), potentially accelerating the rate which populations can adapt to abiotic filtering. Most invasive species found at high altitudes are suggested to have high adaptation ability associated with strong phenotypic adjustment capabilities (Haider *et al.* 2010; Alexander and Levine 2019). This phenotypic adjustment capability may involve both phenotypic plasticity and genetically based trait differentiation (Ghalambor *et al.* 2007; Hoffmann and Sgro 2011; Halbritter *et al.* 2018). For example, many invasive plants are believed to be experience high phenotypic plasticity at the beginning of an invasion, while adaptation lags behind (van Kleunen *et al.* 2010; Molina-Montenegro *et al.* 2016; Hiatt and Flory 2020).

In our field survey, we observed that the MAR of *G. quadriradiata* seeds increased with elevation (Fig. 2F), possibly due to a combination of phenotypic plasticity and genetic differentiation. However, we also found that MAR decreased with elevation in the greenhouse study (Fig. 5D), when seeds from different populations were grown in a shared, non-limiting environment. Mass–area ratio first increased and then decreased with elevation in the field, but it consistently decreased with source elevation in the greenhouse study **[see****Supporting Information—Fig. S1****]**, suggesting that dispersal-related traits are in part determined by plastic responses to environmental conditions. Therefore, we speculate that phenotypic plasticity also plays a considerable role in the invasion dynamics of this species. This is further evidenced by our findings that the MAR of the greenhouse populations was much lower than that in the field **[see****Supporting Information—Fig. S1****]**, MAR of the highest population decreased by 94.10 % while that of the lowest population decreased by 88.50 %, relative to in the field.

This inconsistent variation along elevational gradients between dispersal architecture (especially pappus length) and other dispersal-related traits has also been detected by others (Gravuer *et al.* 2003). It may be caused by environmental variation in mountain ranges that could underlie the effects of inherent genetic differentiation. Environmental variation was suppressed in the greenhouse, thus decreasing phenotypic variation of dispersal-related traits. Along the elevational gradient, the changing environmental conditions caused dispersal-related traits to change in ways that did not reflect the interpopulation genetic differentiation. Therefore, our results indicate that phenotypic plasticity also plays an important role in the expansion of *G. quadriradiata* in mountain ranges. Furthermore, it indicates that mountains could play a key role in slowing down the dispersion of *G. quadriradiata* not only directly by abiotic factors but also indirectly by changing its dispersal-related traits. Therefore, we conclude that variation of dispersal-related traits for this species reflects genetic divergence from low elevation conditions. We further speculate that the trait variation reflects changes in dispersal ability; however, further research will be needed to ascertain this. Regardless, this study provides empirical evidence for the evolution of dispersal-related traits in the process of plant invasion into high mountain ranges.

## Conclusions

Trait variation among populations has been associated with changes in natural selection pressure along elevation (Bode and Dufresne 2019). Long-term adaptation to environmental factors would thus result in genetically based changes in phenotypic traits (Marin *et al.* 2019; Pimpinelli *et al.* 2019). In this study, patterns of genetic differentiation were found to be associated with elevational differences among populations of *G. quadriradiata* in central China. This differentiation was also associated with dispersal-related traits of this species, suggesting that dispersal ability may be genetically based. Moreover, differentiation in MAR between elevational populations in the greenhouse experiment (Fig. 5D; **see****Supporting Information—Fig. S1**) suggests that trait variation may also be partially controlled by phenotypic plasticity, echoing results from other studies (Holtsford and Ellstrand 1992). Our results therefore indicate that genetically based rapid evolution, as well as phenotypic plasticity, play important role in the expansion of *G. quadriradiata* in mountain ranges. Although our results support the idea that mountain ranges can act as natural barriers to plant invasions, the plasticity demonstrated in the greenhouse experiment implies that once acclimated to high elevations, acclimating back to low elevations on the other side will occur quickly and therefore should be cautiously monitored. Further research is needed to link the traits from this study directly to dispersal ability as well as to investigate these patterns across broader geographic gradients.

## Supporting Information

The following additional information is available in the online version of this article—

**Figure S1.** MAR (mass–area ratio) of the four populations in greenhouse experiment and field.

**Table S1.** The random effects of linear mixed-effect models on MAR (mass–area ratio), HSW (weight of one hundred seeds), NSC (Number of mature seed per capitulum), seed length, pappus length and pappus width.

**Table S2.** The random effects of linear mixed-effect models on PPL, *I* and *H*_e_.

plab008_suppl_Supplementary_Materials

plab008_suppl_Supplementary_Data

## Data Availability

The data used in this study are available as **Supporting Information**.

## Notes

**Populations & Communities. Chief Editor:** Jean Burns
