# Supplementary material for: The dispersal-related traits of an invasive plant Galinsoga quadriradiata correlate with elevation during range expansion into mountain ranges
Source: AoB Plants. 2021 Jun 16;13(3):plab008. doi: 10.1093/aobpla/plab008 (PMC8237851; doi:10.1093/aobpla/plab008)
Supplement: plab008_suppl_Supplementary_Materials [file plab008_suppl_supplementary_materials.docx]

**Supplementary material**


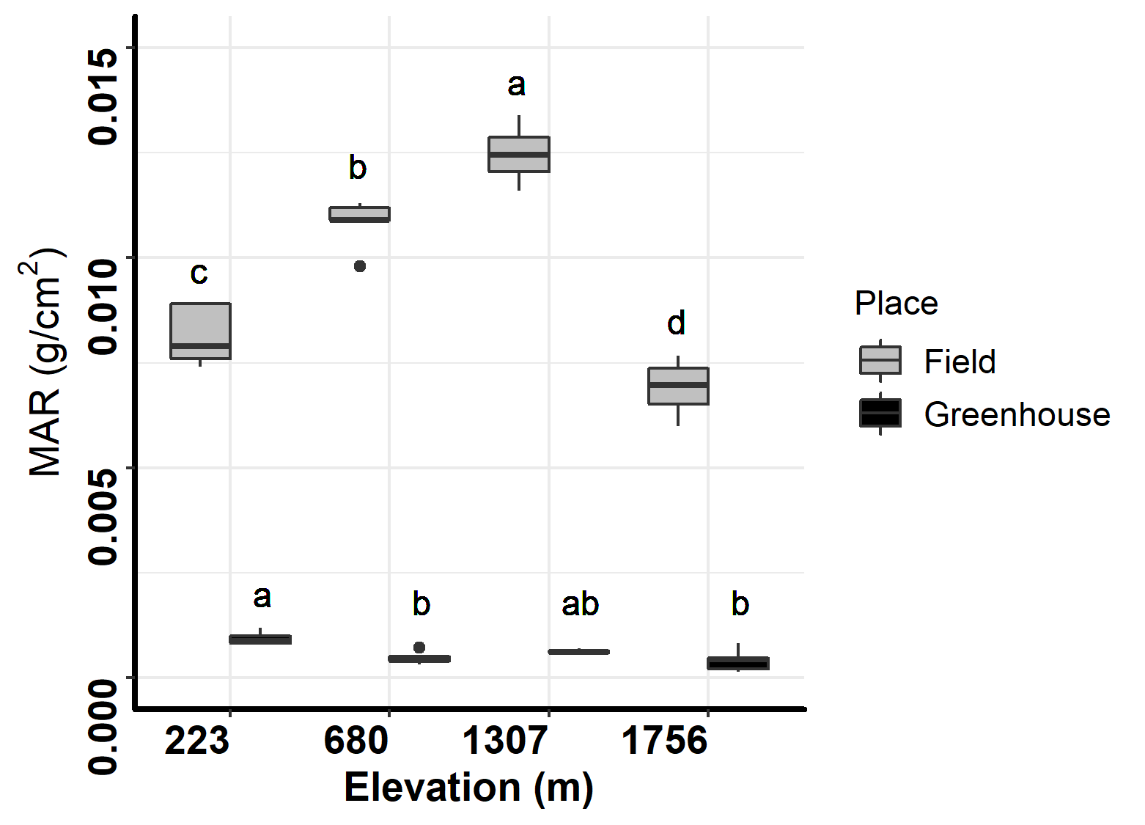


**Fig. S1** MAR of the four populations in greenhouse experiment and field. Different letters indicate significant differences at *p* < 0.05 between the elevational populations in each place.

**Table S1** The random effects of linear mixed effect models on MAR (mass–area ratio), HSW (weight of one hundred seeds), NSC (Number of mature seed per capitulum), Seed Length, Pappus Length, and Pappus width.

| Dependent  variable | Random effects | Variance | Std.Dev. |
| --- | --- | --- | --- |
| MAR | Mountain | 8.10×10^-9^ | 9.00×10^-5^ |
|  | Residual | 8.83×10^-6^ | 0.003 |
| HSW | Mountain | 9.26×10^-7^ | 0.001 |
|  | Residual | 1.13×10^-5^ | 0.003 |
| NSC | Mountain | 0.386 | 0.622 |
|  | Residual | 15.708 | 3.963 |
| Seed Length | Mountain | 0.001 | 0.022 |
|  | Residual | 0.013 | 0.112 |
| Pappus Length | Mountain | 0.003 | 0.053 |
|  | Residual | 0.047 | 0.216 |
| Pappus Width | Mountain | 0.003 | 0.052 |
|  | Residual | 0.16 | 0.4 |

**Table S2** The random effects of linear mixed effect models on PPL, I, and He.

| Dependent  variable | Random effects | Variance | Std.Dev. |
| --- | --- | --- | --- |
| PPL | Mountain | 0.001 | 0.022 |
|  | Residual | 0.001 | 0.029 |
| I | Mountain | 1.3×10^-4^ | 0.011 |
|  | Residual | 2×10^-4^ | 0.016 |
| He | Mountain | 5.9×10^-5^ | 0.008 |
|  | Residual | 1.16×10^-4^ | 0.011 |
